# Advanced breast cancer clinical nursing curriculum: review and recommendations

**DOI:** 10.1007/s12094-016-1530-0

**Published:** 2016-08-03

**Authors:** C. Vila, C. Reñones, T. Ferro, Mª. Á. Peñuelas, M. del Mar Jiménez, Á. Rodríguez-Lescure, M. Muñoz, R. Colomer

**Affiliations:** 1 Oncology Department, Hospital Clínico San Carlos, Madrid, Spain; 2 UME-UCPAL-HADO, Complejo Hospitalario Universitario, Santiago de Compostela, Spain; 3 Direction of Nursing, Institut Català d’Oncologia, L’Hospitalet de Llobregat, Barcelona, Spain; 4 Medical Oncology Department, Hospital Universitario de la Vall d’Hebrón, Barcelona, Spain; 5 Oncology Clinical Management Unit (UGC), Hospital de Jaén, Jaén, Spain; 6 Medical Oncology Department, Hospital General de Elche, Alicante, Spain; 7 Medical Oncology Department, IDIBAPS, Hospital Clínic de Barcelona, Barcelona, Spain; 8 Medical Oncology Department, Hospital Universitario de la Princesa, Madrid, Spain

**Keywords:** Breast neoplasms, Oncology nursing, Case management, Patient navigation, Counselling, Consensus

## Abstract

**Purpose:**

The needs and concerns of patients with advanced breast cancer are changing at every phase of the care intervention. Management and coordination of hospital resources and services are also steadily evolving. The objective of the present expert report is to define a new oncology nursing role specialising in advanced breast cancer, to help guide patients throughout the whole healthcare itinerary.

**Methods:**

A group of eight experts in oncology nursing and medical oncology defined the content index of the curriculum document. A systematic review of bibliography was carried out, and the relevant contents were extracted. Based on these contents and the participants’ experience, recommendations were formulated and validated through a Delphi questionnaire and a participative meeting.

**Results:**

The advanced breast cancer clinical nurse (ABCCN) should develop a clinical, psychosocial role focused on coordinating patients in the healthcare network. The nurse would be in charge of evaluating and supervising the care administered and the healthcare resources used. The ABCCN should be aware and participate in the protocols and available resources, be able to solve conflicts, deal with burn-out signs and have clinical, coaching and team-working abilities. The proposed curriculum provides a specific process for the care of patients, as well as an implementation process.

**Conclusions:**

The ABCCN’s role is crucial to assume the best care and the optimisation of available resources. This review and consensus document provides the required tools for the implementation in hospitals.

## Introduction and objectives

Breast cancer is the most common tumour among women in developed countries [1], and approximately one-third of patients in the clinic have advanced disease [2, 3].

In recent years, there has been much progress in therapeutic options in advanced breast cancer, which has increased the disease control and has significantly contributed to improving prognosis in a substantial proportion of patients. These patients require complex intervention and care, as well as good coordination of all specialists involved. These factors significantly influence on patients’ prognosis and quality of life [4, 5].

NICE guidelines on the management of advanced breast cancer emphasise the need to assess the physical, psychological, social, spiritual and financial needs of patients at some key points in the course of the disease: diagnosis, start of treatment, end of treatment, relapses and end of life [6]. Guidelines include the designation of a healthcare provider assuming the role of “key worker” for each patient and promoting continuous care [6]. Nursing professionals, in particular, have a key role in the management of patients with advanced breast cancer, contributing with their perspective on patients’ experience in different therapeutic and medical situations. In coordination with oncologists specialised in breast cancer, they can accelerate healthcare processes, improve continuous care and minimise symptoms and adverse effects of treatment, as well as help patients cope with fears, improve relationship with their environment, detect situations of particular fragility and achieve quicker interventions.

There is no current consensus document or curriculum in Spain that defines the professional profile, roles or activities of an advanced breast cancer clinical nurse (ABCCN). The purpose of this document is to review and establish a consensus on the activities to be performed by nursing professionals in an advanced breast cancer patients’ care consultation, as well as to turn nursing professionals into key members on multidisciplinary teams and providers of comprehensive support to patients, especially in terms of emotional considerations, health education and follow-up of toxicities. The ABCCN can help achieve better self-care among patients, greater treatment adherence and a decrease in oncological emergencies.

## Methods

This document of recommendations was developed by a multidisciplinary team composed of eight experts in oncology nursing and medical oncology, following the Methodological Manual for the Preparation of Clinical Practice Guidelines in the Spanish National Health System (*Elaboración de Guías de Práctica Clínica en el Sistema Nacional de Salud. Manual metodológico*), which forms part of the Quality Plan of the Spanish Ministry of Health [7].

At the start of the process, a coordinating committee (CC), with three experts, and a recommendation-formulating group (RFG), composed of the members of the CC and five more experts were formed. All of them developed, in an initial face-to-face meeting in December 2014, a content index and a list of clinical questions to be addressed. Subsequently, a systematic literature search was conducted in three databases (MEDLINE, Trip database and ENFISPO). A total of 236 publications were obtained. At the CC’s discretion, a total of 56 publications were prioritised. After reading them, a document answering each clinical question and including potential recommendations was prepared. Next, the recommendations were assessed individually by each RFG member, without any kind of communication or exchange of opinions. Both non-consensus recommendations and consensus recommendations liable to change at the CC’s discretion were debated during a structured participatory face-to-face meeting in July 2015. Recommendations that achieved unanimity (100 % agreement) or consensus (≥80 % agreement) were accepted as definitive. At the end of the process, a total of 147 recommendations (131 by unanimity and 16 by consensus) were validated. The most important recommendations were formally categorised with their level of evidence (LE) and degree of recommendation (DR), according to the modified version of the Scottish Intercollegiate Guidelines Network system [8].

## Results

### Roles and activities of the ABCCN

#### Clinical role

Median survival for advanced breast cancer is ~3 years. During the survival period, the entire set of therapeutic strategies––hormone therapy, chemotherapy, targeted therapies, radiotherapy and palliative care––is applied in different sequences or combinations [9].

Given that throughout the course of the disease, there are constant variations in the type, dose and frequency of treatment, the clinical management of patients with advanced breast cancer should cover their needs at all points in the process, ensuring comprehensive quality care and being based, at all times, on clinical practice guidelines (Agreement: 100 %; LE/DR: 4/D) [10, 11]. The ABCCN should participate in continuous care and in clinical management, providing information and access to diagnosis tests, administering oncological and complementary treatments and managing adverse effects and pain (Agreement: 100 %; LE/DR: 4/D) [12–14]. In any case, the ABCCN should follow updated protocols of therapeutic regimens, thereby avoiding variability in clinical practice with respect to administration of oncological procedures and interventions against the occurrence of toxicities (Agreement: 100 %; LE/DR: √). She/he should also assess clinical parameters for patients with advanced breast cancer, such as comorbidities, performance status, nutrition, pain, general prognosis, toxicities, and symptom management (Table 1).

**Table 1:** Clinical parameters recommended to be evaluated by the ABCCN

Clinical parameters	Agreement (%)	LE/DR
Comorbidities	88	√
ECOG score	88	√
Nutritional considerations	100	√
Pain assessment	100	√
Prognosis	100	√
Current and residual toxicities	100	√
Symptom management	100	√

The ESO–ESMO consensus on advanced breast cancer, developed by the European School of Oncology and the European Society for Medical Oncology, with the objective of improving health outcomes, recommends psychological care and a personalised approach to patients from the very moment they are diagnosed [9]. Along these lines, the expert panel considered that psychological care should be multidisciplinary (Agreement: 100 %; LE/DR: √). The ABCCN should pay attention to psychological considerations for patients and their families throughout the process, and engage in good communication with them, providing appropriate information at each point in the process (Agreement: 100 %; LE/DR: 4/D) [9, 15, 16]. Also, it is recommended that the ABCCN monitors patients’ anxiety levels, so that psychological abnormalities that must be addressed by mental health professionals could be early detected (Agreement: 100 %; LE/DR: 4/D) [17].

As advanced breast cancer is a chronic disease, it is important to highlight the ABCCN’s role as a coach. Coaching tools improve adherence to treatment and control of adverse effects, as well as the patients’ health education assimilation (Agreement: 88 %; LE/DR: √). The ABCCN should apply communication techniques, such as active listening and empathy, and promote patients’ active participation in their health process and autonomy (Agreement: 100 %; LE/DR: 4/D) [14, 18, 19]. In this regard, it is also relevant to offer solid health education.

#### Education and research role

The ESO–ESMO guidelines recommend that all patients with advanced breast cancer receive comprehensive, culturally sensitive, up-to-date and easy-to-understand information about their disease and its management (level of evidence IB) [9]. Patients should understand that, at present, advanced breast cancer is treatable but not curable, although some patients can live with the disease for very long periods of time. Education should be started to teach patients and their families to recognise and treat the different adverse effects of the treatment received (Agreement: 100 %; LE/DR: 4/D) [9, 10]. The ABCCN should teach, advise and guide patients and caregivers with respect to the disease and treatments, as well as to healthy living habits related to diet, exercise, rest and sleep (Agreement: 100 %; LE/DR: 4/D) [9, 10]. In more advanced phases of the disease, she/he must focus education on the management of symptoms, including pain or anxiety, applying the best continuous care available to patients (Agreement: 100 %; LE/DR: √). One suggestion is organising training activities with patients and their families regarding different problems and concerns, which may be previously identified through patient surveys. Holding workshops with them is an effective way to provide specific training, by giving them the chance to share their experiences and concerns among equals and, at the same time, to receive suggestions that may be an opportunity for change (Agreement: 100 %; LE/DR: √). Also, it is important to establish both individual and group education protocols (Agreement: 100 %; LE/DR: √).

The ABCCN can offer training related to the process of advanced breast cancer, not only to patients but also to other health professionals, covering those subjects that each professional requires at her/his care level (Agreement: 100 %; LE/DR: √). The ABCCN can also act as a mentor to students completing their internships in the Oncology unit, focusing on the global vision of the process of advanced breast cancer and recognising her/his role and how the care provided to patients should be (Agreement: 100 %; LE/DR: √). At the same time, she/he should participate in the process of training nursing residents, to ensure the consolidation of advanced practice in the care of patients with advanced breast cancer (Agreement: 100 %; LE/DR: 4/D) [20, 21]. Owing to the chronic nature of the disease, there is direct contact with primary care. Thus, it would be useful for the nursing staff at health centres to know the protocols for action, the management of symptoms, pain and adverse effects, the handling of technical devices such as reservoirs and peripherally inserted central catheters and palliative patient care (Agreement: 100 %; LE/DR: √). The expert panel came to a consensus on offering training to other non-oncology units that may care for patients with advanced breast cancer (Agreement: 88 %; LE/DR: √), as well as to oncologists, since their knowledge of the nursing care needs of patients is often limited (Agreement: 88 %; LE/DR: √).

In the course of the teaching and clinical work, the ABCCN should make use of resources that may serve as a vehicle for conveying accurate and comprehensible information to patients regarding their disease, treatment, adverse effects and sequelae (Agreement: 88 %; LE/DR: √). These resources may be presented on paper (manuals and glossaries of terms, newspapers, individualised and standardised medication regimens and lists of adverse effects and their management), involve telematics (telephone consultation, interactive digital platforms, websites, patient associations and single-subject workshops) or healthcare services (social work, psycho-oncology, nutrition, physiotherapy and primary care) (Agreement: 100 %; LE/DR: √).

#### Case management role

Patients with advanced breast cancer require continuous care from different health professionals who meet their needs whenever they arise. Therefore, the role of the ABCCN as case manager has also been established [14]. The expert panel agreed that the ABCCN should supervise and evaluate the different healthcare options and services offered to patients and their families (Agreement: 88 %; LE/DR: 4/D) [22, 23]. The ABCCN should also take care of logistical considerations such as moving up appointments, fostering the coordination of different appointments in a single visit, shortening waiting times and managing transfers, all to minimise the psychosocial impact on patients and those around them (Agreement: 100 %; LE/DR: 4/D) [22, 23]. Finally, the ABCCN must act in collaboration with the multidisciplinary team, engaging in direct communication with patients and families and providing information on the steps in the care process (Agreement: 100 %; LE/DR: 4/D) [22, 23].

### Professional profile

While no evidence was found with respect to how much experience is required to perform the role of ABCCN, the participating experts agreed a minimum of 2 years of continuous experience with oncology patients to be able to perform the role of ABCCN with certain minimum guarantees (Agreement: 100 %; LE/DR: √). Various publications were evaluated to find out the ideal training, and while some of them favour having a specific certification to practise this role, others believe that a degree in health science or simple experience is enough [23–27]. To achieve the skills, knowledge and attitudes required to work efficaciously, efficiently and effectively, it is advisable to have specific training in oncology (Agreement: 100 %; LE/DR: 4/D), mainly in clinical, case management and counselling aspects (Agreement: 100 %; LE/DR: √).

There is no oncology nursing specialty within the current panorama of qualified training in Spain. However, it is important to design a face-to-face or remote continuous education programme for ABCCN (Agreement: 100 %; LE/DR: √) that covers all considerations related to comprehensive care of patients, their physical and psychosocial considerations and those of their families (Agreement: 100 %; LE/DR: 4/D) [23]. The ABCCN should receive specific training in lines of treatment, care and treatment protocols, adverse effects, pain management, palliative care, communication skills and counselling (Agreement: 88 %; LE/DR: 4/D) [23, 28]. At the same time, she/he should receive training in research, promoting and fostering the performance of this role and in the different clinical trials in which patients are likely to participate (Agreement: 88 %; LE/DR: √). Consequently, the ABCCN can also act as healthcare collaborator in clinical research (Agreement: 100 %; LE/DR: 4/D) and as research nurse, acting as principal investigator in nursing care studies (Agreement: 88 %; LE/DR: 4/D) [29].

Also, from the expert panel, scientific societies are advised to drive the creation of accreditations to assess the nursing curriculum, taking into account matters such as training, teaching, research or care provided (Agreement: 100 %; LE/DR: √). The ABCCN also requires a broad knowledge of the treatment protocols for advanced breast cancer and the operations at the centre where she/he works, as well as being informed of research activities. The ABCCN should know about and participate on breast cancer committees, be familiar with the resources available to offer help to patients both inside and outside of the hospital and know about patient associations, support groups and different complementary therapies (Agreement: 100 %; LE/DR: 3/D) [30].

### Competencies

The Spanish Society of Oncology Nursing (SEEO, according to its Spanish acronym) has been aware of the need to promote the establishment of nursing competencies in cancer patients and research within this framework. In this regard, a specific competency framework for oncology nurses was established at the second SEEO conference in 2005 [30]. The expert panel also formulated recommendations on the ABCCN’s competencies (Table 2).

**Table 2:** Competencies of the ABCCN

Group	Recommendation	Agreement (%)	LE/DR
Patient assessment	Perform the patient assessment systematically (both objective and subjective data) and record it within the conceptual framework adopted in the department	100	√
	Analyse the data and draw conclusions for subsequent care	100	√
	Share the data with the rest of the team	100	√
	Skilfully manage the capacity to evaluate, diagnose and treat patients, within the professional boundaries of nursing	100	√
Clinical management	Diagnose (identify problems), plan interventions, execute (including interdependent and autonomous nursing interventions) and perform a final assessment. This process requires a continuous evaluation of the care plan and makes it possible to decide whether the objectives of the care plan established have been reached	100	√
	Offer comprehensive care to patients, based on scientific evidence	100	√
	Involve patients’ families and/or loved ones in care	100	√
	Duly inform patients so that they are capable of making appropriate decisions at every point in their disease	88	√
	Know what the healthcare system offers, to be able to inform patients and help them move within this framework	100	√
	Coordinate the care offered to patients with the rest of the therapeutic team and ensure that care is continuous	100	√
	Know the ethical and legal principles that should govern nursing practice and incorporate them into care	100	√
Counselling/coaching	Assess, at their first appointments, patients’ and families’ capacities for and attitudes towards learning about lifestyle throughout the disease process	100	√
	Bear in mind patients’ values and beliefs and consider the coping mechanisms at their disposal, to deal with the disease at each stage	100	√
	Prepare patients by proposing short- and medium-term objectives	100	√
	Mobilise patients’ personal resources and provide tools to achieve short- and medium-term objectives	100	√
	Support patients and families at different points in the experience of living with cancer	100	√

### Skills

To effectively assume the role of ABCCN, certain specific skills should be acquired. The ABCCN establishes a relationship of confidence with their patients, thus facing the possibility of being emotionally affected by their patients’ situation. She/he should have emotional self-knowledge and recognise her/his emotions and how they can affect in a high-risk department (Agreement: 100 %; LE/DR: √). The ABCCN must always be conscious of her/his mood, as this could influence her/his behaviour (Agreement: 100 %; LE/DR: √). Also, the ABCCN should be capable of exercising self-control and self-motivation and of channelling her/his own emotions towards an objective, focusing on goals and trying to overcome obstacles (Agreement: 100 %; LE/DR: √). She/he should be capable of experiencing empathy, recognising the emotions of others, knowing how to interpret what is happening to patients, understanding them and performing an assertive analysis of specific situations (Agreement: 100 %; LE/DR: √). Finally, the ABCCN should be competent in social skills, valuing interpersonal relationships as essential to human well-being and having certain values, such as tolerance, communicative abilities and knowledge of how to persuade people, negotiate with them, calm them down and reconcile situations of conflict (Agreement: 100 %; LE/DR: √).

The ABCCN interacts with a multidisciplinary team, patients and their families and caregivers, as well as with healthcare and social systems. To do this, she/he must possess conflict resolution skills. The ABCCN should approach the conflict by applying a set of rules, through basic consensus between the parties (Agreement: 100 %; LE/DR: √). When there is no clear consensus, the ABCCN needs skills and experience to help approach different positions (Agreement: 100 %; LE/DR: √). Problem resolution should be done through a participatory process in which all parties jointly determine what the problem is and come to a resolution (Agreement: 100 %; LE/DR: √). The ABCCN should be capable of negotiating and assessing whether a conflict is resolved and whether the parties involved are satisfied (Agreement: 100 %; LE/DR: √).

Teamwork is crucial to approach cancer management with unified criteria [31]. Regarding teamwork skills, it was agreed that the ABCCN should be capable of proposing, analysing, consulting and implementing methods that enhance the healthcare process together with the rest of the members of the interdisciplinary team (Agreement: 100 %; LE/DR: √). With the objective of improving the relationships between the people involved in the different processes, the ABCCN should be capable of agreeing upon shared objectives for the healthcare plan and of actively participating in meetings, sessions and other activities that foster multidisciplinary work (Agreement: 100 %; LE/DR: √).

### Healthcare process for advanced breast cancer patients

**Fig. 1:**
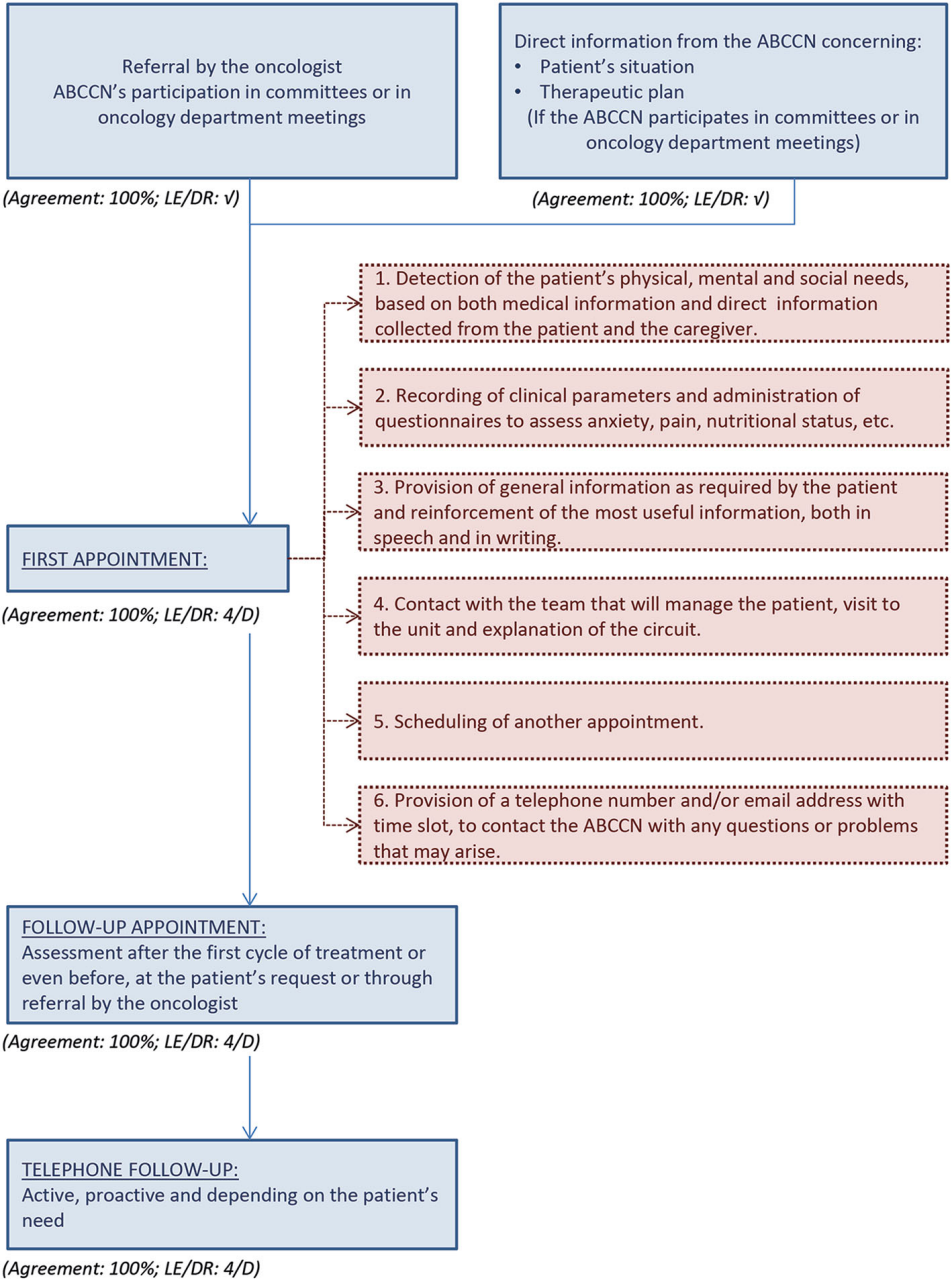
Decision-making and assistance algorithm for advanced breast cancer patients

It is necessary to establish a healthcare track for patients with advanced breast cancer, to keep them from not visiting the ABCCN owing to a lack of information (Agreement: 100 %; LE/DR: √). Initially, access of patients to the ABCCN should be convenient and hassle-free, through her/his own visit schedules. An initial visit is required when patients are diagnosed and referred from medical consultation, and successive appointments must then be scheduled, depending on the assessment and patients’ needs. The participating experts established recommendations and came to a consensus on a circuit for how to develop advanced breast cancer patients’ care, as described in Fig. 1.

Patients with advanced breast cancer may have many and varied needs throughout the process. It is, therefore, necessary to establish the circumstances that call for intervention by an ABCCN. Diagnosis requires an initial intervention for assessment and a first contact to evaluate therapies, symptoms, adverse effects, anxiety and pain control (Agreement: 88 %; LE/DR: √). Also, the ABCCN should facilitate contact with other professionals, either at the level of hospital care (psycho-oncology, rehabilitation and diagnostic support services—radiology, pathology, nuclear medicine, among others) or at the level of primary care, liaison nurses, palliative home care, social workers, etc. (Agreement: 100 %; LE/DR: √).

### Implementation process

Key nurses or liaison nurses, who are focused on cancer either in general or on particular types, are relatively new in the Spanish healthcare system. Therefore, there is insufficient experience on which criteria should be evaluated when implementing this type of role, although the literature points to an achievement of greater effectiveness and efficiency in patient care [32]. The expert panel recommended assessing the implementation of a key position such as the ABCCN if the following criteria are considered:

- Reducing the impact of hospital fragmentation caused by the application of the therapeutic plan, and offering a coherent and simple environment for patients.
- Eliminating the gaps that may arise in a multidisciplinary care system.
- Improving the patient experience by eliminating or reducing negative situations that could be prevented or mitigated.
- Promoting patient empowerment throughout the healthcare process, especially in stages of greater vulnerability such as diagnosis, recurrence and treatments (Agreement: 100 %; LE/DR: √).

**Fig. 2:**
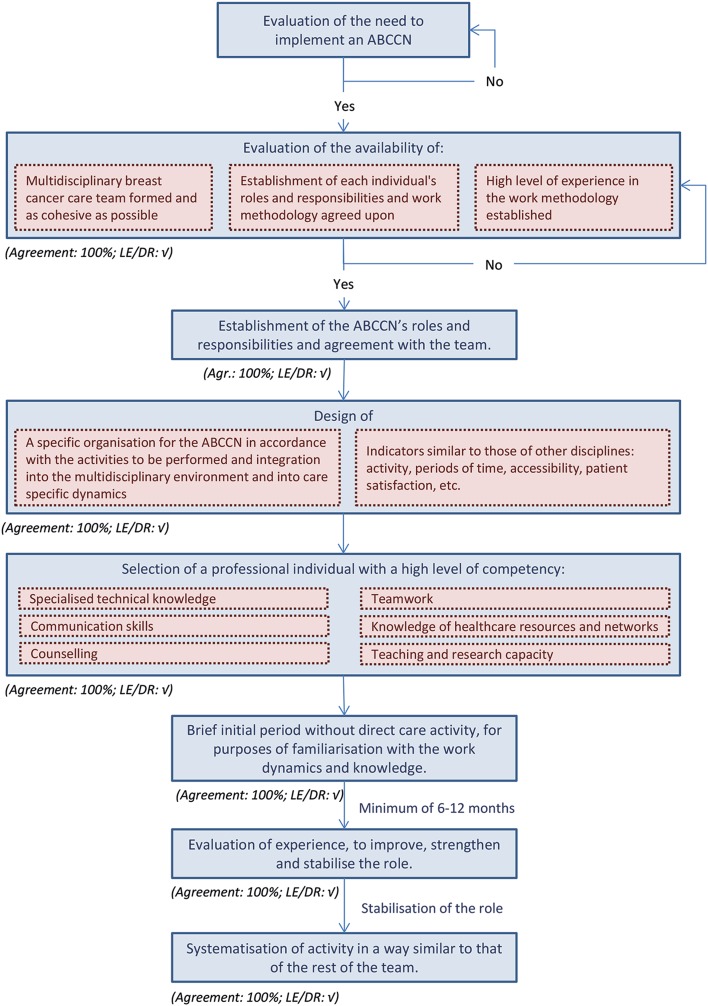
Implementation process of the ABCCN’s position

The first step in the implementation process on which the authors of this document have come to a consensus (Fig. 2) is the evaluation of the need for an ABCCN. It is advisable to drive this implementation in a coordinated manner with institutional support and evaluation (Agreement: 100 %; LE/DR: √).

For the designation of the ABCCN, it is advisable to have a description of the position, based on the professional profile, competencies and skills (Agreement: 88 %; LE/DR: √), in addition to a specific methodology for assessing competencies (assessment of professional CV, knowledge, abilities and aptitudes) (Agreement: 100 %; LE/DR: √).

It is appropriate to be careful with the method of initiation at the institution. Introducing the ABCCN to other healthcare professionals may be a novel event, especially at centres that do not have similar previous experience in other areas, and it should ideally be done by managers or corresponding authorities, through a formal introduction to all the departments and units involved in the field of action of the ABCCN (Agreement: 100 %; LE/DR: √). At the same time, the start of this new role and its objectives, responsibilities and location should be communicated by the usual means (intranet, for instance) (Agreement: 100 %; LE/DR: √). It would be beneficial to have previously worked with the multidisciplinary team, introducing the ABCCN and agreeing upon their objectives and roles (Agreement: 100 %; LE/DR: √). Also, it is advisable to agree with this team upon a gradual introduction that allows for effective learning (Agreement: 100 %; LE/DR: √).

Recording the activity of the ABCCN is also considered to be important, and the expert panel agreed that this should be included in the institution’s usual schedules (face-to-face or telephone visits, telephone emergency care, telemedicine, etc.) (Agreement: 100 %; LE/DR: √). At the same time, it is also advisable to define some ABCCN activity indicators (Agreement: 100 %; LE/DR: √), although there are other activities associated with this role that require time but are difficult to quantify, such as case management (Agreement: 100 %; LE/DR: √). Some examples of healthcare activity indicators could be: attributable periods of time in the healthcare process, number of emergencies owing to treatment toxicity, number of central catheters in treatments that carry a risk of chemical phlebitis, adherence to oral treatments and referrals through screening to nutrition, psycho-oncology or oncogeriatrics departments, etc.

## Conclusions

The care of cancer patients has undergone substantial changes in recent years. Their physical and psychological needs should be handled appropriately through continuously provided care, and their demands should be met in all phases of the disease. In the case of ABC, there is a wide range of therapeutic options, support measures, required resources as well as a considerable number of specialists who are involved in the management. This contributes to the complex nature of these patients’ follow-up.

The SEOM Handbook on Continuous Oncology Care recommends coordinating and optimising all available healthcare and social resources, fostering the integration of all healthcare levels and building comprehensive oncology care, with the aim of improving patient’s quality of life [33]. The ESMO–ESO consensus on advanced breast cancer recommended including a nurse specialised in oncology (if possible, breast cancer) in the multidisciplinary team in charge of managing patients with advanced breast cancer [9], clearly showing the need to add a key figure in the care of these patients.

Key nurses in cancer are referred to by many names, depending on the country in which they practise nursing and the roles they perform: Healthcare Coordinators, Patient Navigators, Health Coaches, Care Managers, Nursing Case Managers, Oncology Nursing Navigators, Oncology Patient Navigators and Key Oncology Nurses are just some of these names [23, 34]. This curriculum has established the definition and roles of the ABCCN, as well as the skills and professional profile which, in any case, may be developed by professionals who are also in charge of other roles or specialists in other areas.

The roles of nurses specialised in oncology should include assessment of individual patient needs, education, coordination, communication and support provided to patients, implementation of effective transitions in the course of the disease and evaluation of the consequences for patients, families and the organisation [34]. The clinical role of ABCCN has paid special attention to psychological considerations, healthcare education and coaching for patients and families and has focused on managing symptoms, pain and anxiety. Likewise, the roles of case workers have also been established previously [14, 19, 23, 35, 36], and this consensus document recommends that the ABCCN takes logistical measures to minimise the psychosocial impact on patients and their loved ones, such as moving up appointments, fostering coordination of appointments in a single visit and shortening waiting times.

This document establishes several requirements in terms of training and personal skills for the ABCCN and recommends the provision of specific continuing education in lines of treatment, healthcare protocols, adverse effects, pain management, palliative care, care for terminally ill patients, communication, counselling and clinical research skills.

Following the course set in 2005 by the SEEO at its second conference [30], where a specific competency framework for oncology nurses was established, the authors of this document formulated and validated a set of recommendations on the ABCCN’s competencies with respect to patient assessment, clinical management, counselling and coaching.

In summary, this consensus document will be a useful tool to improve the care and quality of life of patients with advanced breast cancer throughout the entire care process. It presents expert agreements and recommendations on the care provided by specialised nurses to patients with advanced breast cancer. It also establishes the profile and roles of the ABCCN, the healthcare process in which she/he participates and the resources required to perform her/his activity.

The present manuscript provides an implementation algorithm that can be useful for those centres without previous experience in creating specialised nursing roles or with difficulties in implementing such positions as an ABCCN. Finally, it recommends evaluating the need for reducing the impact of fragmentation of services, offering patients a simpler and more coherent environment, eliminating the gaps that may arise in multidisciplinary care, improving the patient experience and promoting patient empowerment throughout the healthcare process before starting implementation.
